# When Common Tumours Hide in Uncommon Places: Paediatric Sacral Ewing Sarcoma With Metastases

**DOI:** 10.7759/cureus.94789

**Published:** 2025-10-17

**Authors:** Audrey R Rumhumha, Nabeela Kazee, Mantsane Mokone

**Affiliations:** 1 Radiology, University of Witwatersrand, Johannesburg, ZAF

**Keywords:** case report, ebstein barr virus, ewing sarcoma, hepatic metastases, magnetic resonance imaging, metastases, paediatric oncology, sacrum, small round blue cell tumour

## Abstract

Ewing’s sarcoma is a highly aggressive malignant small round blue cell tumour that typically affects the long bones and axial skeleton in children and adolescents. Sacral involvement is uncommon, often leading to diagnostic delays due to nonspecific symptoms. We report a case of a nine-year-old girl presenting with chronic back pain and weight loss, eventually found to have a large sacral mass with hepatic metastases. Imaging revealed a destructive presacral tumour with soft tissue extension, and histopathology confirmed Ewing’s sarcoma with EWSR1 rearrangement. This case highlights the diagnostic challenges of rare anatomical sites, emphasises the value of multimodality imaging, and reinforces the importance of including Ewing’s sarcoma in the differential for persistent paediatric back pain.

## Introduction

The Ewing’s sarcoma family of tumours (ESFT) is the second most common primary malignant bone tumour in children and adolescents after osteosarcoma [1,2]. Typical sites include long bone diaphyses, pelvis, and ribs. Sacral involvement is distinctly uncommon, accounting for fewer than 5% of spinal Ewing’s sarcomas [3,4], and frequently leads to diagnostic delay due to subtle or non-specific symptoms.

The differential diagnosis for sacral masses in children includes chordoma, neuroblastoma, lymphoma, rhabdomyosarcoma, and sacrococcygeal teratoma, many of which demonstrate overlapping radiological appearances. Histopathological and molecular analysis, therefore, remain essential for confirmation.

This report describes a rare case of sacral Ewing’s sarcoma with synchronous hepatic metastases in a nine-year-old girl, further notable for EBV seropositivity. The case highlights the importance of early suspicion, comprehensive imaging, and integrated diagnostic correlation to improve outcomes in paediatric back pain presentations.

## Case presentation

A nine-year-old girl presented with an eight-to-nine-month history of progressive lower back and gluteal pain initially attributed to “growing pains.” The pain gradually worsened, limiting mobility and resulting in a 5-kg weight loss. Neurological examination was normal; a firm, tender sacral mass was palpable. A structured summary of the clinical and diagnostic timeline is presented in Table 1.

**Table 1:** Clinical Timeline Summary.

Months	Event/Investigation
0	Onset of intermittent lower back pain.
3–6	Progressive worsening of pain with reduced mobility and weight loss. During this period, the patient had multiple consultations at local general practitioners and primary healthcare facilities.
7	Referred to a level 1 district hospital where pelvic radiographs were obtained. Findings prompted referral to a tertiary centre.
8	Presentation to the orthopaedic department at a tertiary hospital. A firm sacral mass was palpated. Radiographs were followed by MRI for further characterisation of a suspected malignant lesion in view of the protracted symptoms, weight loss, and destructive sacral changes seen on imaging.
9	CT abdomen demonstrated multiple hepatic metastases.
10	CT-guided biopsy confirmed Ewing’s sarcoma with EWSR1 gene rearrangement.

Laboratory test results revealed elevated inflammatory markers (ESR 40 mm/hr, lactate dehydrogenase (LDH) 2206 U/L) and mild hyponatraemia. EBV serology demonstrated positive EBNA IgG and VCA IgG with negative VCA IgM, consistent with past EBV infection; no EBER in situ hybridisation was performed. Results are shown in Tables 2-4.

**Table 2:** Laboratory Investigations. ↑ above reference range; ↓ below reference range. CRP: C-reactive protein; ESR: erythrocyte sedimentation rate; ALT: alanine transaminase; AST: aspartate transaminase; ALP: alkaline phosphatase; GGT: gamma-glutamyl transferase; LDH: lactate dehydrogenase.

Test	Result	Reference Range	Units	Comment
Blood Chemistry				
Sodium	129 ↓	136 – 145	mmol/L	Mild hyponatraemia
Chloride	98	98 – 107	mmol/L	Normal
Bicarbonate	23	23 – 29	mmol/L	Normal
Anion gap	17 ↑	9 – 16	mmol/L	Mildly raised
Urea	4.3	1.4 – 5.7	mmol/L	Normal
Creatinine	49	28 – 57	µmol/L	Normal
Inflammatory markers C-reactive protein (CRP)	69 ↑	<10	mg/L	Elevated: inflammatory response
Full Blood Count				
White cell count	9.07	3.90 – 10.20	×10⁹/L	Normal
Red cell count	5.63 ↑	3.80 – 5.40	×10¹²/L	Mildly raised
Haemoglobin	14.5	10.3 – 15.5	g/dL	Normal
Haematocrit	0.464 ↑	0.310 – 0.460	L/L	Slightly high
MCV	82.4	77.1 – 91.5	fL	Normal
MCH	25.8	25.8 – 31.7	pg	Lower end of normal
MCHC	31.3 ↓	33.0 – 35.1	g/dL	Mildly reduced
Red cell distribution width	13.9	11.6 – 14.8	%	Normal
Platelet count	245	180 – 440	×10⁹/L	Normal
MPV	11.0	7.0 – 11.4	fL	Normal
ESR	40 ↑	0 – 10	mm/hr	Elevated: non-specific inflammation
Additional Chemistry				
Calcium	2.37	2.12 – 2.57	mmol/L	Normal
Magnesium	0.88	0.66 – 0.95	mmol/L	Normal
Inorganic phosphate	1.31	1.00 – 1.80	mmol/L	Normal
Uric acid	0.29	0.12 – 0.30	mmol/L	Normal
Liver Function Tests				
Total protein	68	57 – 80	g/L	Normal
Albumin	38	29 – 42	g/L	Normal
Total bilirubin	3 ↓	5 – 21	µmol/L	Slightly low (not clinically significant)
Conjugated bilirubin	2	0 – 5	µmol/L	Normal
ALT	16	5 – 25	U/L	Normal
AST	35	0 – 41	U/L	Normal
ALP	147	69 – 325	U/L	Normal
GGT	74 ↑	4 – 22	U/L	Elevated
LDH	2206 ↑	110 – 295	U/L	Markedly elevated

**Table 3:** Epstein–Barr Virus (EBV) Serology.

Test	Result
EBV Nuclear Antigen (EBNA) IgG	Positive
Viral Capsid Antigen (VCA) IgG	Positive
Viral Capsid Antigen (VCA) IgM	Negative

**Table 4:** Interpretation of EBV Serological Patterns. EBNA IgG: EBV Nuclear Antigen Immunoglobulin G, VCA IgG: Viral Capsid Antigen Immunoglobulin G, VCA IgM: Viral Capsid Antigen Immunoglobulin M Summary Interpretation for this patient: EBNA IgG: Positive, VCA IgG: Positive, VCA IgM: Negative. This serological pattern is consistent with past EBV infection (latent phase), not acute primary infection.

VCA IgM	EBNA IgG	Interpretation
Negative	Negative	No evidence of EBV exposure
Negative	Positive	Evidence of past exposure and infection with EBV
Positive	Negative	Compatible with primary EBV infection
Positive	Positive	Suggestive of EBV reactivation

**Figure 1:**
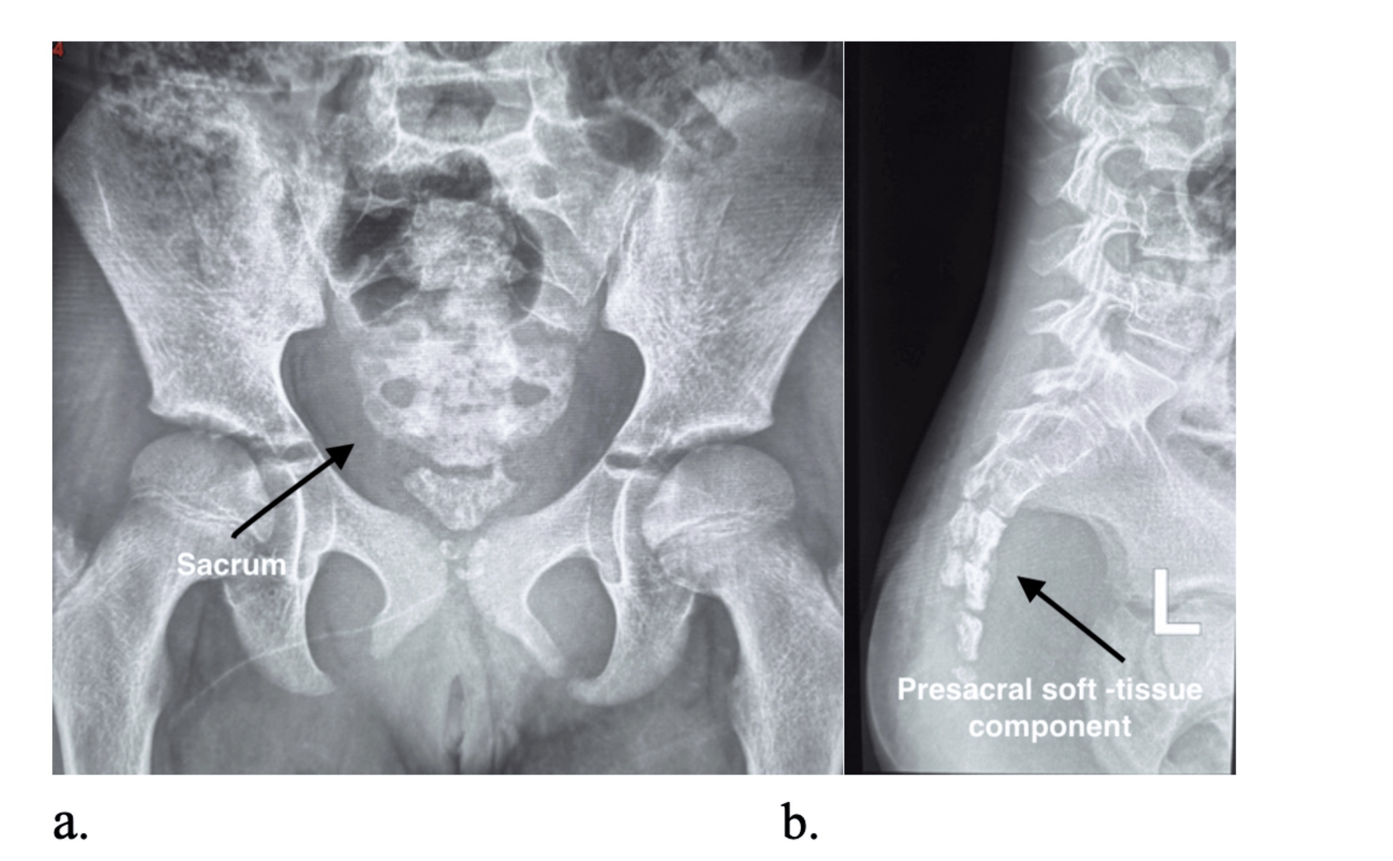
(a) Anteroposterior and (b) Lateral Pelvic Radiographs Demonstrating a Destructive Expansile Lesion Centered at the Sacrum (S2–S3) (Black Arrows). These features are characteristic of an aggressive sacral neoplasm and highlight the importance of correlating radiographic findings with MRI for full local staging.

**Figure 2:**
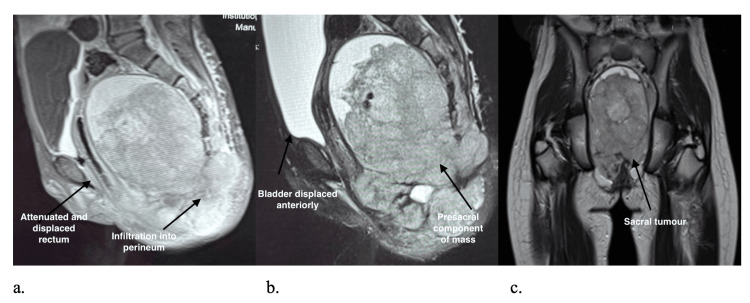
MRI Pelvis. (A) Sagittal Post-Contrast T1-Weighted Image, (B) Sagittal T2-Weighted Image, and (C) Coronal T2-Weighted Image. Demonstrating a large heterogeneous sacral mass with marrow infiltration and presacral soft-tissue extension (arrows). The lesion exhibits cortical destruction, marrow infiltration, and a soft-tissue component extending anteriorly into the presacral and bilateral ischioanal regions. There is effacement of the neural foramina and mass effect on the rectum. The imaging features are characteristic of an aggressive primary sacral tumour and, in this context, are consistent with Ewing’s sarcoma. MRI provides superior delineation of marrow involvement, neural encasement, and soft-tissue extent compared with other modalities.

**Figure 3:**
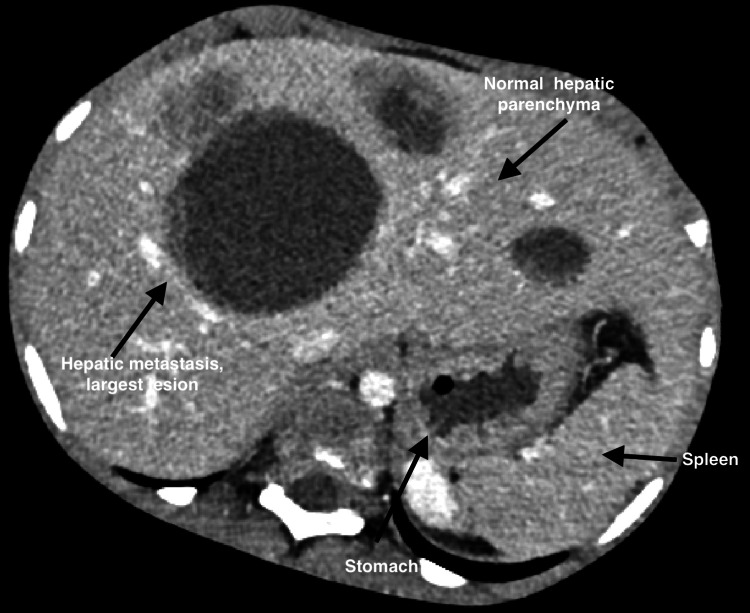
Contrast-Enhanced Axial Slice CT Scan of Abdomen. It is showing multiple hypodense hepatic metastases. A large dominant metastatic lesion is seen in the right hepatic lobe, with smaller lesions visible bilaterally. The stomach and spleen appear preserved. No intrahepatic biliary dilatation or ascites is noted. These findings confirm haematogenous dissemination from the primary sacral Ewing’s sarcoma and illustrate the classic CT appearance of hepatic metastases; well-defined, low-attenuation lesions relative to enhancing liver parenchyma.

Radiographs showed an inhomogeneous sacral mass with lytic destruction and cortical expansion at S2-S3 (Figure 1). MRI demonstrated a large heterogeneous presacral mass (98.7 × 149.4 × 143.2 mm) with cortical destruction, marrow infiltration, bilateral ischioanal extension, and foraminal effacement (Figure 2). A CT abdomen demonstrated multiple hypodense hepatic metastases (Figure 3).

Differentials included chordoma, teratoma, rhabdomyosarcoma, lymphoma, and Ewing’s sarcoma. A CT-guided biopsy confirmed sheets of small round blue cells with strong membranous CD99 positivity. FISH detected EWSR1 rearrangement, confirming Ewing’s sarcoma (Table 5).

**Table 5:** Histopathology, Immunohistochemistry and Molecular Findings. Summary: Histology showed a classic small round blue cell tumour morphology. Immunohistochemistry demonstrated CD99 and FLI1 positivity, with negative myogenic and neuroendocrine markers. FISH confirmed EWSR1 gene rearrangement in 50% of cells. Final integrated diagnosis: Ewing sarcoma of the sacral region.

Category	Finding	Details/Interpretation
Histology	Tumour type	Undifferentiated paediatric small round blue cell tumour
Histology	Morphology	Neoplastic proliferation of small round blue cells with hyperchromatic nuclei and scant cytoplasm arranged in nests separated by fibrous stroma. No rosette formation or germ cell tumour features. No osteoid deposition.
Histology	Invasion	Tumour infiltrates skeletal muscle. No lymphovascular or perineural invasion identified. No necrosis seen.
Initial Differential Diagnosis		Ewing sarcoma and Desmoplastic small round cell tumour
Immunohistochemistry (Set 1)	CD99	Positive membranous staining
	AE1/3	Focal nuclear staining in isolated tumour cells
	Desmin	Very focal cytoplasmic staining (skeletal muscle)
	Myogenin	Negative
	Synaptophysin	Negative
	Chromogranin	Negative
	LCA	Negative in tumour, reactive lymphocytes highlighted
Immunohistochemistry (Set 2)	EMA	Negative
	ERG	Negative
	FLI1	Positive nuclear staining
	Vimentin	Positive in tumour cells and stroma
Molecular Testing (FISH)	Target	EWSR1 (22q12) gene rearrangement
	Method	Fluorescence in situ hybridisation (Vysis break-apart probe)
	Result	Positive — 50% of cells screened showed rearrangement
	Interpretation	Confirms EWSR1 gene rearrangement, supportive of Ewing sarcoma
Final Diagnosis		Undifferentiated paediatric small round blue cell tumour, consistent with Ewing sarcoma

The patient commenced VAC/IE chemotherapy. PET/CT was not performed; given the clear evidence of hepatic metastases on CT, its omission would not have altered management. Owing to the extent of the disease, curative surgery was precluded. The patient remains on systemic therapy with interval imaging for reassessment.

## Discussion

Diagnostic challenges and imaging

Ewing’s sarcoma accounts for approximately 10-15% of paediatric bone malignancies [1,2]. Spinal involvement occurs in around 10% of cases, with the sacrum being the least frequent site [3,4]. Nonspecific presentations, most often back pain, lead to prolonged diagnostic intervals. In this case, symptoms persisted for nearly nine months before cross-sectional imaging was obtained.

The diagnostic pathway typically progresses from plain radiography, which may be normal early on, to MRI for local staging and CT for cortical assessment and biopsy planning [4]. PET/CT is increasingly used for systemic staging and treatment response assessment. In our patient, radiographs demonstrated sacral destruction, MRI defined the soft-tissue and neural foraminal extension, and CT established hepatic metastases.

The differential diagnosis for destructive sacral lesions in children includes chordoma (midline, often calcified), teratoma (fat or calcification, especially in neonates), rhabdomyosarcoma (soft-tissue origin), and lymphoma. Ewing’s sarcoma should always be considered, particularly when imaging demonstrates an aggressive lytic lesion with soft-tissue extension.

Histopathology and molecular findings

Histology revealed a uniform proliferation of small, round blue cells with hyperchromatic nuclei and scant cytoplasm. Immunohistochemistry showed strong CD99 and FLI1 positivity, excluding myogenic, lymphoid, and neuroendocrine origins. Detection of the EWSR1-FLI1 fusion remains the molecular hallmark of Ewing’s sarcoma [5]. Demonstration of EWSR1 rearrangement by FISH confirmed the diagnosis in this patient.

Integration of histopathological, immunohistochemical, and molecular data is vital in atypical locations such as the sacrum, where morphology alone may be non-specific.

Prognostic and therapeutic considerations

Poor prognostic factors include metastases at diagnosis, tumour size >8 cm, axial or pelvic location, and poor chemotherapy response [5-7]. Elevated LDH, as in this patient, reflects high tumour turnover and correlates with aggressive biology. Hepatic metastases are rare and portend advanced disease with a poor prognosis.

Treatment is multimodal: neoadjuvant multi-agent chemotherapy, followed by local control with surgery or radiotherapy, and adjuvant therapy [1,2,5]. Sacral tumours often necessitate radiotherapy to preserve neurological function while achieving local control.

EBV seropositivity: possible relevance

Positive EBV serology (EBNA IgG and VCA IgG, VCA IgM negative) indicated prior exposure, not acute infection. EBV seropositivity is common by adolescence and is unlikely to be causative in Ewing’s sarcoma. Previous studies have found no EBV genomic material in tumour cells [8,9].

However, EBV may modulate host immune responses or cytokine profiles, potentially influencing tumour microenvironment dynamics. An experimental study suggested that EBV microRNAs can target EWSR1, raising the possibility of viral-host molecular crosstalk [10].

There are also isolated case reports describing EBV reactivation in association with Ewing sarcoma, sometimes in the setting of immune dysregulation or haemophagocytic lymphohistiocytosis [11].

While the clinical significance remains unproven, awareness of such mechanisms may inform future research, particularly in high-prevalence regions.

Clinical implications and education

This case reinforces several important principles for clinicians: 1) persistent or progressive back pain in children should prompt early imaging, ideally MRI, even when neurological findings are absent. 2) Multimodal diagnostic integration is crucial in atypical anatomical sites. 3) EBV seropositivity, though likely incidental, raises interesting biological questions meriting further exploration.

By documenting the coexistence of hepatic metastases and EBV seropositivity at initial presentation, an exceedingly rare combination, this case adds to the limited global literature on unusual manifestations of Ewing’s sarcoma.

## Conclusions

Sacral Ewing’s sarcoma is a rare and diagnostically challenging entity. Its deep pelvic location, non-specific symptoms, and late neurological signs often delay diagnosis. Persistent paediatric back pain, particularly when associated with systemic symptoms such as weight loss, warrants prompt and thorough investigation with early MRI. This case underscores the importance of an integrated multimodal diagnostic approach combining imaging, histopathology, immunohistochemistry, and molecular testing. The presence of hepatic metastases at diagnosis denotes advanced disease and poor prognosis.

Although EBV seropositivity likely reflects prior exposure, it introduces an intriguing biological variable. While EBV is not recognised as causative in Ewing’s sarcoma, ongoing research into possible immunomodulatory interactions may yield valuable insights. Ultimately, this report reinforces a key take-home message: in children, persistent back pain is never benign until proven otherwise. Early imaging, particularly MRI, can be lifesaving.
